# All-cause and cause-specific mortality among transgender and gender diverse people: a nationwide cohort study in Australia

**DOI:** 10.1016/j.lanwpc.2026.101813

**Published:** 2026-02-09

**Authors:** Karinna Saxby, Sasha Bailey, Dennis Petrie, Brendan J. Nolan

**Affiliations:** a Melbourne Institute: Applied Economic & Social Research Faculty of Business and Economics, The University of Melbourne, Room 5.55, Level 5, FBE Building, 111 Barry Street, Carlton, Victoria, 3010, Australia; b Centre for Health Economics, Monash Business School, Monash University Victoria, Australia; c Centre for Youth Mental Health, University of Melbourne, Victoria, Australia; d National Centre for Excellence in Youth Mental Health, Orygen, Parkville, Australia; e Trans Health Research Group, Department of Medicine, University of Melbourne, Victoria, Australia; f Department of Diabetes and Endocrinology, Princess Alexandra Hospital, Woolloongabba, Queensland, Australia; g Faculty of Medicine, University of Queensland, Brisbane, Queensland, Australia

**Keywords:** Transgender, Gender diverse, Mortality, External cause, Cardiovascular disease, Cancer

## Abstract

**Background:**

Transgender and gender diverse (‘trans’) people may be at increased risk of mortality, particularly from external causes, however, large-scale, population-based evidence remains scarce. This study aims to document all-cause and cause-specific mortality among trans people.

**Methods:**

We source administrative data on healthcare and death records (2012–2023) from all Australians aged 15 years and above. Individuals identified as trans (initiated gender-affirming hormone therapy) were matched to the general population who visited a General Practitioner during the same period. Cox proportional hazard models were used to estimate all-cause and cause-specific mortality risk for trans people, with models estimated separately for people who were assigned female sex at birth (AFAB) and people who were assigned male sex at birth (AMAB). Inverse probability weights were applied to balance comparators on month-year and age at entry. Results were additionally stratified by age (15–24, 25–39, 40–59, and ≥60 years).

**Findings:**

A total of 19,347 trans people AMAB (mean age 36.2 years; median follow-up 3.7 years) and 9713 trans people AFAB (mean age 25.4 years; median follow-up 2.8 years) were matched with 9,879,037 general population males (mean age 44.4; median follow-up 11.3 years) and 10,282,651 general population females (mean age 44.9; median follow-up 11.4 years), respectively. All-cause mortality was significantly higher for trans people AMAB [HR = 3.89 (95% CI 33.65; 4.14)] and trans people AFAB [HR = 9.03 (95% CI 6.90; 11.83)]. For trans people AFAB, cause-specific mortality was elevated for cardiovascular disease [HR = 16.39 (95% CI 8.56; 31.37)], suicide [HR = 11.73 (95% CI 6.94; 19.80)], external causes [HR = 8.95 (95% CI 5.69; 14.09)], and cancer [HR = 7.62 (95% CI 4.61; 12.61)]. For trans people AMAB, cause-specific mortality was elevated for cancer [HR = 5.12 (95% CI 4.67; 5.61)], suicide [HR = 4.02 (95% CI 3.12; 5.18)], external causes [HR = 2.78 (95% CI 2.31; 3.35)], and cardiovascular disease [HR = 2.60 (95% CI 2.22; 3.05)]. Older trans people had more pronounced excess risk from cancer and cardiovascular disease. For trans people AFAB, excess mortality from suicide and external causes increased with age, whereas for trans people AMAB relative risks were higher in young and middle adulthood.

**Interpretation:**

In this nationwide cohort study, trans Australians experienced substantially elevated mortality risk. Tailored policy responses are needed to address premature mortality in trans populations.

**Funding:**

University of Melbourne McKenzie Fellowship (2025MCK182); the 10.13039/501100000925National Health and Medical Research Council (2008956); 10.13039/100008717Viertel Charitable Foundation; University of Melbourne Faculty Research Grant (2025FRG19).

**Research in Context**

**Evidence before this study**

PubMed was searched from 1 January 2000 to October 8, 2025, using a structured search strategy restricted to terms appearing in the title or abstract fields. The search terms were: (“transgender” [ti] OR “transsexual” [ti] OR “gender diverse” [ti] OR gender incongruence” [ti] OR “gender dysphoria” [ti]) AND (“mortality” [ti] OR “cause-specific mortality” [ti] OR “all-cause mortality” [ti]) AND Humans [MeSH] NOT (review [pt] OR systematic [sb] OR meta-analysis [pt]). This search returned nine studies. After screening titles and abstracts, seven studies were identified as being directly relevant to the research question; i.e., investigating either all-cause or cause-specific mortality among transgender and gender diverse (‘trans’) people relative to the general population.

Five studies were based on specific cohorts or non-representative samples. Analyses using US Veterans Health Administrative data found elevated suicide mortality but lower all-cause mortality among trans people (identified based on ICD codes for gender dysphoria; and ∼75% assigned male at birth (AMAB)), relative to cisgender males. A follow-up study using subgroup of this Veterans dataset who had a depression diagnosis had elevated all-cause and cardiovascular-related mortality. Two other US studies on a sample of individuals with private health insurance reported elevated all-cause mortality among trans people AMAB but not for trans people assigned female at birth (AFAB). A study of primary care data in England reported higher external cause mortality for trans people AFAB relative to general population females but not for trans people AMAB relative to general population males.

Two population-based European studies were identified. One study documented elevated mortality risk among trans people using hormone therapy treatment in the Netherlands compared to modelled mortality risk in the general population, particularly for external causes, and for HIV and lung cancer among trans people AMAB. A Danish psychiatric registry study found that compared to the general population, individuals identified with gender incongruence had elevated all-cause and suicide mortality risk but noted that these differences were non-significant for trans people identified through administrative records.

Taken together, findings are mixed and vary by subpopulation and cause of death. Moreover, in part due to sample size and data limitations, there are limited assessments on mortality risk for different trans populations, including evidence on how cause-specific mortality varies by sex at birth and over the life course.

**Added value of this study**

Using linked, nationwide Australian administrative data (2012–2023), we provide the largest population-level assessment to date of all-cause and cause-specific mortality among trans people, with weighted general-population comparators, and stratification by sex at birth and age. In this cohort (n = 20,190,748; 204,261,719 person-years), all-cause mortality was substantially higher for both trans people AFAB (HR 9.03, 95% CI 6.90; 11.83) and people trans people AMAB (HR 3.89, 95% CI 3.65; 4.14) versus their matched counterparts. For trans people AFAB, the largest excesses were seen for cardiovascular disease, suicide, external causes, and cancer, whereas for trans people AMAB, excess mortality was greatest for cancer, suicide, and external causes. These results indicate there is significant heterogeneity in mortality risk within trans populations that previous studies have been underpowered to detect.

**Implications of all the available evidence**

Trans populations experience premature mortality relative to the general population, albeit these patterns vary by sex assigned at birth and age. These findings highlight the need for urgent and tailored policy response focused on cardiovascular disease and cancer prevention (risk-factor management, screening uptake, and continuity of care), alongside strategies to prevent deaths from external causes, including suicide. Policy responses should consider the social factors that perpetuate these health inequities.

## Introduction

Transgender and gender diverse (hereafter, ‘trans’) people have gender identities that differ from their assigned sex at birth. Global estimates suggest that 0.5–4.5% of adults and 2.5–8.4% of children and adolescents identify as trans.^1^

Trans people face significant health inequities, including higher rates of physical and mental ill-health, cardiovascular disease, asthma, and certain cancers.^2^, ^3^, ^4^, ^5^, ^6^, ^7^, ^8^ There is also significant heterogeneity in medical comorbidities within trans populations, shaped by differences in sex assigned at birth, medical affirmation (for those who desire and subsequently seek it), and interpersonal and structural discrimination.^9^^,^^10^

Theoretical frameworks such as ‘minority stress theory’ posit that these health inequalities are, in part, driven by exposure to stigma-related stressors and that this chronic stress produces cumulative psychological and physiological burden.^11^ A large body of research demonstrates that chronic exposure to external stressors—such as discrimination, social exclusion, and interpersonal violence—has measurable biological effects. These include dysregulation of the central nervous system responses, increased systemic inflammation, and alterations in immune function; all of which are implicated in the development of cardiovascular disease, metabolic disorders, and certain cancers.^12^ Long-term exposure to social and economic disadvantage has also been associated with accelerated biological ageing (including telomere shortening and allostatic load) consistent with the ‘weathering’ hypothesis which has been postulated to explain racial disparities in health^13^^,^^14^ It is plausible to speculate that this ‘weathering’ hypothesis may have generalisability to trans populations given trans people are commonly socially and economically disempowered (e.g. due to high rates of workplace discrimination, mistreatment and exclusion)^15^ and housing insecurity stemming from discrimination from family, co-habitants, and rental agencies.^16^ In accordance with these theories, empirical studies document that trans populations are more likely to report modifiable secondary risk factors associated with increased risk of mortality, including higher rates of regular (e-) cigarette, alcohol, and other drug use; poor dietary behaviours, poor sleep, and lower levels of physical activity, compared to the cisgender population.^17^^,^^18^^,^^19^

Despite increased recognition on health inequities experienced by trans populations, few studies have investigated all- and cause-specific mortality and to date most studies have been based on non-representative samples or surveys.^20^^,^^21^ Moreover, in part due to sample size and data limitations (e.g., representativeness and selection bias inherent in data/methods used for identifying trans sub-groups) and heterogeneity in populations investigated, these sparse studies have provided mixed findings. For instance, an analysis of US private health insurance data found higher all-cause mortality risk among trans people who were assigned male at birth^g^ (AMAB) compared with general population males, but not among trans people who were assigned female at birth (AFAB) compared with general population females.^20^ Evidence from the Netherlands indicates that trans people AMAB face slightly elevated risks of cancer and cardiovascular-related mortality relative to cisgender males, whereas no significant differences in these outcomes were observed for trans people AFAB relative to cisgender females.^22^ Altogether, uncertainties remain.

To our knowledge, there have only been two large-scale, population-based investigations. One study documented elevated mortality risk among 4568 trans people (mean follow-up: 12.6 years; n = 1641 (36%) AFAB; n = 2927 (64%) AMAB) using hormone therapy treatment the Netherlands compared to the general population, particularly for external causes [standardised mortality ratio (SMR) 2.7 (95% CI 1.8; 3.7) for trans people AMAB and SMR 3.3 (95% CI 1.2; 6.4) for trans people AFAB].^22^ Substantial differences were also observed in HIV-related mortality and lung cancer among trans people AMAB relative to general population males.

The other, based in Denmark, found that compared to the general population, individuals identified with gender incongruence in a National Psychiatric Research Register (mean follow-up: 5.7 years; n = 1784 (48%) AFAB; n = 1975 (52.5%) AMAB) had elevated all-cause and suicide mortality risk [Incidence Rate Ratio (IRR) 2.0 (95% CI 1.7; 2.4) and IRR 3.5 (95% CI 2.0; 6.3), respectively], but noted that these differences were non-significant for trans people identified through administrative records.^23^ While these studies provide important insights, by focussing on individuals seeking care for gender dysphoria or who were hospitalised, they may overrepresent those experiencing psychological distress.

The present study is the largest population study to date of mortality among trans individuals and the first from Australia. The aim of this study is to provide a contemporary, robust assessment on whether trans people in Australia experience higher all-cause and cause-specific mortality than similar, likely cisgender, individuals from the general population.

## Methods

### Data

The data for this analysis comes from Australia's Person Level Integrated Data Asset (PLIDA), an individual-level linked dataset which combines information from the Australian population Census and various administrative data sources including healthcare records, social security records, income, and death records.^24^ Healthcare records capture publicly subsidised out-of-hospital healthcare services and prescription medications for all Australian citizens and permanent residents (provided under Australia's universal health insurance scheme, Medicare). Death records contain information on date and cause of death. For this study, we use data from April 2012 to December 2023 (the most recent and complete data available at the time of writing).

### Study cohort

We applied a retrospective cohort study and included all people aged 15 and above who accessed gender-affirming hormone therapy and presumed cisgender people who visited a General Practitioner (GP) between 2012 and 2023. We restricted the cohort to people aged 15 years and over because, in Australia, gender-affirming hormone therapy is typically initiated from 15 to 16 years, consistent with the Australian Standards of Care and paediatric endocrine guidelines.^25^

### Exposure

Following previous literature, we identified trans people using information on sex markers and records of filling prescription medication for gender-affirming hormone therapy.^26^ Specifically, individuals who ever filled a prescription for testosterone and had a current or previous female sex marker were classified as trans AFAB and individuals who ever filled a prescription for oestradiol and had a current or previous male sex marker were classified as trans AMAB. In Australia, gender-affirming care is delivered through a combination of mainstream primary care and specialist services. Access to gender-affirming hormone therapy requires a prescription from a medical practitioner and is subsidised through the Pharmaceutical Benefits Scheme for eligible residents. Care pathways vary by state but typically involve assessment by clinicians experienced in trans health, with initiation of gender-affirming hormone therapy most commonly occurring from after 15–16 years of age.^27^ Most jurisdictions require multidisciplinary input in those aged under 18 years, whereas the informed consent model is commonly utilised in those aged 18 years and above.^27^

### Outcomes

The primary outcome was all-cause mortality between April 2012 and December 2023, with person-time calculated from the index date until month-year of death or end of follow-up (December 2023). The index date was defined as the month-year of first gender-affirming hormone therapy prescription for trans individuals and the month-year of first GP visit for the general population comparators. We selected GP attendance as a matching criterion because it represents a near-universal point of contact with the Australian healthcare system and provides a comparable indicator of healthcare engagement around the index date. For expediency, the control populations are referred to as general population males and general population females, although we cannot establish that none of these people are trans.

Cause-specific mortality was classified according to the International Statistical Classification of Diseases and Related Health Problems, Tenth Revision (ICD-10). Cause-specific mortality was examined for four major categories: suicide; external causes; cardiovascular disease (defined as deaths due to ischaemic heart disease, cerebrovascular disease, and other circulatory disorders); and cancer (defined as malignant neoplasms).

Deaths from external causes were classified according to the ICD-10; namely, alcohol-related causes, drug- and poisoning-related deaths, and unintentional injuries. All ICD-10 codes are provided in the Supplementary SM1.

### Statistical analyses

To construct comparable cohorts, we estimated inverse probability weights for the general population using age and month-year of index date as covariates. This weighting procedure reweighted the general population to have the same distribution of age and calendar time of ‘entry’ as each of the trans groups. As a result, the weighted general population reflects the age structure of the trans cohorts, not that of the general population.

We then described the characteristics of the trans and matched general population cohorts and used Kaplan–Meier curves to compare survival across groups.

Cox proportional hazards regression models were fitted to estimate hazard ratios (HRs) and 95% confidence intervals (CIs) for all-cause and cause-specific mortality. For cause-specific analyses, deaths from other causes were treated as censored events (i.e., competing risks were not explicitly modelled). Analyses were stratified by sex assigned at birth (i.e., AFAB vs AMAB) and further by age group at treatment initiation (15–24, 25–39, 40–59, and 60+ years). These age bands were selected to align with the World Health Organisation definition of ‘youth’ as people aged 15–24 years,^28^ while adult categories were further divided to reflect major shifts in baseline risk for cardiovascular disease, cancer, and other age-related outcomes. These divisions also ensured sufficient sample size within each stratum to maintain statistical precision.

We additionally assessed the proportional hazards assumption using Schoenfeld residuals and found evidence of non-proportionality. Subsequently, as a sensitivity analyses, we estimated extended Cox models allowing the effect of trans status on mortality risk to vary over time.^29^

In the baseline analysis, we did not adjust for individual-level characteristics such as socioeconomic background, rurality, or migration history, as these factors may lie on the causal pathway between gender identity and mortality. For example, exclusion from labour market opportunities are well documented among trans people.^30^^,^^31^ Adjusting for such characteristics would therefore constitute over-adjustment and bias estimates toward the null by blocking part of the mechanism through which health inequalities emerge. Nevertheless, as a robustness check, we explored how results varied when weighting the general population to be similar to the trans population with respect to their electorate of residence at index date.

As an additional robustness check, we also explored an alternative specification in which trans people AFAB were compared to general population males and trans people AMAB were compared to general population females, once more weighting based on age and month-year of index date.

All analyses were conducted in Stata version 17. No primary data collection was undertaken. Analyses were performed under the PLIDA data-sharing agreement with the Australian Bureau of Statistics.

This study is reported according to STROBE guidelines.

### Ethics approval

This study was approved by the University of Melbourne Human Research Ethics Committee (Project ID 29421). Because this study used de-identified administrative data accessed through the Australian Bureau of Statistics’ DataLab, individual informed consent was not required under the National Statement on Ethical Conduct in Human Research.

### Role of the funding source

Funding bodies had no role in study design, data collection, data analysis, data interpretation, or writing of the report.

## Results

The analytic sample included 20,190,748 individuals (49% AMAB) followed up for 204,261,719 person-years. This included 9713 trans people AFAB (total 32,342 person-years) and 19,347 trans people AFAB (total 88,072 person-years). Descriptives for the unweighted sample are provided in the Supplementary SM2. Briefly, before weighting, at entry trans people were younger than their cisgender sample (19.50 years younger for trans AFAB compared to general population females and 8.25 years younger for trans AMAB compared to general population males).

The weighted sample descriptive statistics for trans people and their general population counterparts are presented in Table 1. As expected, given our weighting strategy, the weighted general population groups have similar mean ages to the trans cohorts because they were reweighted to match the trans cohorts’ age distribution.

**Table 1:** Descriptive statistics for weighted sample.

	Trans people AFAB (n = 9713)	General population females (n = 10,282,651)	Trans people AMAB (n = 19,347)	General population males (n = 9,879,037)
Age entry, mean, years	25.40	25.45	36.15	36.19
Follow-up, median, years	2.83	2.83	3.67	4.00
Person-years, total	32,342	34,578,818	88,072	46,823,454
Age group entry, years:				
Age 15–24, %	65.47	49.70	40.71	19.70
Age 25–39, %	25.80	46.29	26.43	49.19
Age 40–59, %	6.56	3.80	17.60	23.78
Age 60+, %	2.17	0.20	15.26	7.34
Death – any cause, %	0.61	0.07	5.51	1.48
Death – external, %	0.23	0.03	0.61	0.23
Death – suicide, %	0.19	0.02	0.33	0.09
Death – cardiovascular disease, %	0.10	0.01	0.82	0.33
Death – cancer, %	0.16	0.02	2.52	0.51

Notes: Trans = Transgender and gender diverse; AMAB = Assigned male at birth; AFAB = Assigned female at birth. Descriptive statistics shown are weighted and represent a pseudo-population rather than raw sample counts. General population are those who visited a general practitioner at the same time and are weighted using inverse probability weights so that distribution of observed covariates pertaining to ‘age and month-year of start’ is balanced between groups. Unweighted sample is provided in Supplementary SM1. Standardised mean differences for age at entry were <0.01 in both AFAB vs general population females and AMAB vs general population male matched comparisons, indicating negligible differences between groups after matching.

During follow-up, there were 59 deaths among trans people AFAB (0.61%) and 1066 deaths among trans people AMAB (5.51%). This share was higher than their matched general population counterparts; 0.07% and 1.48% respectively. A higher proportion of all deaths in trans AFAB were from external causes (22 deaths, 37% of all deaths) than for trans people AMAB (118 deaths, 11% of all deaths).

### All-cause and cause-specific mortality

**Fig. 1:**
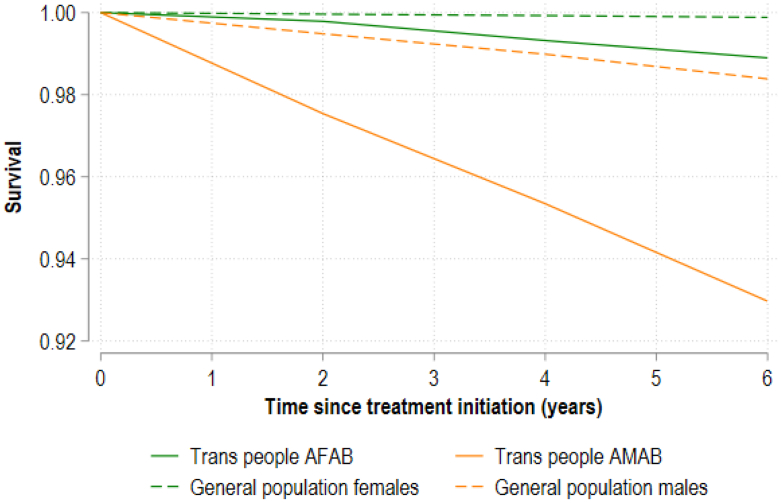
**Cumulative survival in trans people AFAB and trans people AMAB compared with general population males and general population females.** Notes: Trans = Transgender and gender diverse; AMAB = Assigned male at birth; AFAB = Assigned female at birth. Kaplan–Meier survival curves estimated using inverse probability weights to balance general population comparators by age and month-year at treatment initiation. Treatment defined as first gender-affirming hormone therapy prescription for trans people and first General Practitioner (GP) visit for general population.

Kaplan–Meier survival curves demonstrated clear differences in overall survival between trans cohorts and their corresponding matched general population cohort (Fig. 1). Survival among trans people AFAB was slightly lower than that of general population females, while survival among trans people AMAB was markedly lower than that of general population males. Differences between groups widened progressively with time.

At six-years follow-up, estimated survival probabilities were 98.90% for trans people AFAB and 99.88% for general population females; equivalent to an additional 9.87 deaths per 1000 trans people AFAB than the similar general population females.

The corresponding survival probabilities were 92.97% for trans people AMAB and 98.39% for general population males; equivalent to an additional 54.14 deaths per 1000 trans people AMAB than the similar general population males.

Cox proportional hazards models indicated that both trans people AFAB and trans people AMAB had substantially elevated all-cause mortality compared with their general population counterparts (Table 2). The HR for all-cause mortality was 9.03 (95% CI 6.90; 11.83) among trans people AFAB compared with general population females, and 3.89 (95% CI 3.65; 4.14) among trans people AMAB compared with general population males.

**Table 2:** Cause specific Hazard Ratios in trans people AFAB compared with general population females and trans people AMAB compared with general population males.

	Trans people AFAB compared to general population females					Trans people AMAB compared to general population males				
	Full sample	15–24 years	25–39 years	40–59 years	60+ years	Full sample	15–24 years	25–39 years	40–59 years	60+ years
All-cause	9.03∗∗∗ [6.90; 11.83]	3.98∗∗∗ [2.04; 7.77]	6.96∗∗∗ [3.69; 13.14]	3.42∗∗∗ [1.86; 6.29]	2.01∗∗ [1.32; 3.04]	3.89∗∗∗ [3.65; 4.14]	2.54∗∗∗ [1.78; 3.62]	3.09∗∗∗ [2.24; 4.26]	1.86∗∗∗ [1.56; 2.23]	1.966∗∗∗ [1.83; 2.11]
Cardiovascular disease	16.39∗∗∗ [8.56; 31.37]	N/A	19.80∗∗∗ [5.89; 66.58]	N/A	2.94∗∗ [1.35; 6.34]	2.60∗∗∗ [2.22; 3.05]	1.92 [0.33; 11.01]	1.87 [0.61; 5.79]	1.535∗ [1.02; 2.31]	1.18 [0.99; 1.41]
Cancer	7.62∗∗∗ [4.61; 12.61]	N/A	N/A	3.06∗∗ [1.33; 7.02]	1.66 [0.87; 3.17]	5.12∗∗∗ [4.67; 5.61]	1.05 [0.16; 6.92]	0.97 [0.24; 3.96]	1.98∗∗∗ [1.51; 2.61]	2.76∗∗∗ [2.49; 3.05]
External	8.95∗∗∗ [5.69; 14.09]	6.85∗∗∗ [3.41; 13.79]	8.33∗∗∗ [3.37; 20.59]	9.08∗∗∗ [2.85; 28.99]	N/A	2.78∗∗∗ [2.31; 3.35]	2.68∗∗∗ [1.82; 3.96]	4.29∗∗∗ [2.95; 6.25]	2.13∗∗∗ [1.44; 3.16]	1.14 [0.77; 1.70]
Suicide	11.73∗∗∗ [6.94; 19.80]	9.47∗∗∗ [4.16; 21.55]	11.51∗∗∗ [4.54; 29.18]	19.99∗∗∗ [4.81; 83.01]	N/A	4.02∗∗∗ [3.12; 5.18]	4.02∗∗∗ [2.58; 6.26]	6.22∗∗∗ [3.94; 9.82]	2.68∗∗ [1.47; 4.88]	1.54 [0.65; 3.67]

Notes: Trans = Trans and gender diverse; AMAB = Assigned male at birth; AFAB = Assigned female at birth. General population are those who first visited a General Practitioner (GP) at the same time and are weighted using inverse probability weights so that distribution of observed covariates pertaining to ‘age and month-year of start’ is balanced between groups. N/A = not applicable (few deaths in the trans population).

∗∗∗ p < 0.001, ∗∗ p < 0.05, ∗ p < 0.1.

Cause-specific analyses showed that the pattern of elevated mortality differed by sex assigned at birth. For trans people AFAB, the highest excess risks were for cardiovascular disease (HR 16.39 (95% CI 8.56; 31.37)), suicide (HR 11.73 (95% CI 6.94; 19.80)), external causes (HR 8.95 (95% CI 5.69; 14.09)), and cancer (HR 7.62 (95% CI 4.61; 12.61)). For trans people AMAB, mortality risks were greatest for cancer (HR 5.12 (95% CI 4.67; 5.61)), followed by suicide (HR 4.02 (95% CI 3.12; 5.18)), external causes (HR 2.78 (95% CI 2.31; 3.35)), and cardiovascular disease (95% CI 2.22; 3.05)).

Age-stratified analyses showed that elevated mortality risks were evident across all age groups but differed in magnitude by cause and sex at birth. Among trans people AFAB, the highest relative risk observed was for suicide among those aged 40–59 years (HR 19.99 (95% CI 4.81; 83.01)) and for cardiovascular disease among those aged 25–39 years (HR 19.80 (95% CI 5.89; 66.58)). Among trans people AMAB, the largest observed mortality risks were for those aged 25–39 years, particularly for suicide (HR 6.22 (95% CI 3.94; 9.82)) and external causes (HR 4.29 (95% CI 2.95; 6.25)).

Although baseline proportional hazards models (Table 2) suggested higher average relative mortality among trans people AFAB compared with trans people AMAB, models allowing the effect of trans status to vary over time revealed important differences in the timing of excess risk (Table 3). Baseline hazard ratios indicated that, relative to the weighted general population, trans people AMAB experienced similar all-cause mortality risk [HR 5.69 (95% CI 5.14; 6.30)] compared to trans people AFAB [HR 5.21 (95% CI 3.06; 8.86)], but risk varied with time since cohort entry for each trans group. Among trans people AFAB, relative all-cause mortality risk increased with time since cohort entry [interaction with log (time): HR 1.01 (95% CI 1.00; 1.02)]. Cause-specific analyses indicated that this pattern was primarily driven by cancer mortality, for which the relative hazard increased over follow-up [interaction with log (time): HR 1.02 (95% CI 1.00; 1.04)]. Conversely, among trans people AMAB, relative all-cause mortality risk declined over time [interaction with log (time): HR 0.99 (95% CI 0.99; 0.99)]. Reductions in relative risk were most pronounced for suicide mortality [interaction with log (time): HR 0.99 (95% CI 0.98; 1.00)].

**Table 3:** Time varying cause specific Hazard Ratios in trans people AFAB compared with general population females and trans people AMAB compared with general population males.

	Trans people AFAB compared to general population females		Trans people AMAB compared to general population males	
	Baseline hazard ratio	Trend in hazard ratio	Baseline hazard ratio	Trend in hazard ratio
All-cause	5.21∗∗∗ [3.06; 8.86]	1.01∗∗ [1.00; 1.02]	5.69∗∗∗ [5.14; 6.30]	0.99∗∗∗ [0.99; 0.99]
Cardiovascular disease	5.37∗ [1.26; 22.90]	1.03 [1.00; 1.05]	3.21∗∗∗ [2.46; 4.19]	1.00 [0.99; 1.00]
Cancer	3.23∗ [1.16; 9.05]	1.02∗ [1.00; 1.04]	7.66∗∗∗ [6.60; 8.88]	0.99∗∗∗ [0.99; 0.99]
External	6.74∗∗∗ [2.85; 15.95]	1.01 [0.99; 1.03]	4.07∗∗∗ [2.99; 5.53]	0.99∗∗ [0.99,1.00]
Suicide	5.45∗∗ [1.90; 15.61]	1.02 [1.00; 1.05]	7.17∗∗∗ [4.71; 10.92]	0.99∗∗ [0.98; 1.00]

Notes: Baseline hazard ratio is hazard ratio at origin. Trend in hazard ratio refers to log(time since origin). Trans = Trans and gender diverse; AMAB = Assigned male at birth; AFAB = Assigned female at birth. General population are those who first visited a General Practitioner (GP) at the same time and are weighted using inverse probability weights so that distribution of observed covariates pertaining to ‘age and month-year of start’ is balanced between groups. N/A = not applicable (few deaths in the trans population).

∗∗∗ p < 0.001, ∗∗ p < 0.05, ∗ p < 0.1.

Mortality risk was still elevated, and if anything, more pronounced, when additionally matching trans people to a general population residing in the same electorate of residence (Supplementary SM3). Excess mortality additionally persisted when trans people were compared to a matched general population with the opposite sex at birth (Supplementary SM4) however the relative risks for cause-specific mortality and magnitude of effects somewhat differed relative to the baseline model. For example, compared to general population males, trans people AFAB still exhibited the greatest relative excess in cardiovascular mortality (HR 6.62 (95% CI 3.53; 12.43)) however the relative risk for suicide and external cause mortality reduced (to 3.90 (95% CI 2.42; 6.28) and 2.37 (95% CI 1.55; 3.63), respectively). For trans people AMAB compared to general population females, cancer mortality was still significant (5.89 (95% CI 5.36; 6.46) but the relative risk for suicide and external causes was larger (14.01 (95% CI 10.74; 18.28) and 6.75 (95% CI 5.59; 8.15), respectively).

## Discussion

In this nationwide cohort study, trans Australians experienced substantially elevated all-cause mortality compared with their general population counterparts. Excess risks were evident for both trans people AFAB and trans people AMAB, though the risks varied by cause of death. For trans people AFAB, the largest excesses were seen for cardiovascular disease, suicide, external causes, and cancer, whereas for trans people AMAB, excess mortality was greatest for cancer, suicide, and external causes, with more modest elevations for cardiovascular disease. Age-stratified analyses revealed differing risk patterns by sex assigned at birth. Among trans people AFAB, relative risks for suicide and external-cause mortality increased with age. In contrast, among trans people AMAB, suicide and external-cause mortality followed a ‘U-shaped’ pattern, with the greatest excesses observed in young and middle-aged adults. For both trans people AMAB and trans people AFAB, cancer and cardiovascular disease remained important contributors in older adulthood. Our study also demonstrated that excess mortality risk among trans Australians was not static over time; among trans people AFAB, relative all-cause mortality risk increased with time since cohort entry, a pattern driven primarily by rising cancer mortality in later follow-up. In contrast, among trans people AMAB, relative all-cause mortality declined over time, with the most pronounced reductions observed for suicide mortality.

These findings broadly align with previous international evidence on documenting elevated mortality risk in trans populations,^20^, ^21^, ^22^, ^23^^,^^32^ but extend this literature by providing the first estimates for Australia and by demonstrating marked heterogeneity by sex at birth, cause of death, and age.

For example, an analysis of privately insured trans people in the US observed higher all-cause mortality among trans AMAB compared to general population males but not for trans people AFAB compared to general population females,^20^ whereas we observed elevated all-cause risks in both groups. However, because this sample was restricted to individuals with private health insurance, it may suffer from selection bias; particularly as trans people in the US are disproportionately underinsured.^33^

Similarly, while we observed elevated mortality risk for cancer and cardiovascular disease in both trans people AMAB and trans people AFAB, a Dutch study only reported slightly elevated risks for cancer and cardiovascular disease for trans people AMAB relative to general population males (SMR 1.3 (95% CI 1.0; 1.6) and 1.4 (95% CI 1.0; 1.8), respectively), but non-significant differences for these conditions among trans people AFAB relative to general population females. Their study also documented elevated suicide mortality for trans people AMAB, relative to general population males, but not for trans people AFAB relative to general population females (SMR 3.1 (95% CI 1.8; 4.7) and SMR 2.8 (95% CI 0.6; 6.8), respectively). Notably, the sample included 1641 trans people AFAB and 2927 trans people AMAB, compared with 9713 and 19,347 in our cohort, respectively. To this end, it is possible that our larger sample size offered more power to detect these differences. In addition, the use of standardised mortality ratios rather than hazard ratios may partly explain differences in findings. SMRs rely on external reference rates and average risk over follow-up, and may attenuate relative effect estimates compared with internal time-to-event models, particularly when excess risk varies by age or time since cohort entry.^34^ Our results therefore suggest larger excess risks from cancer, cardiovascular disease and suicide mortality among both trans people AFAB and trans people AMAB than previously documented.

The declining relative mortality risk observed among trans people AMAB over follow-up, particularly from suicide, may reflect social experiences relative to medically transitioning. For example, studies suggest that experiences of discrimination, economic disruption, and psychological distress among trans people AMAB often peak around the period of medical and social transition, but attenuate over time.^35^^,^^36^ In contrast, for trans people AFAB, time-varying increases in relative mortality—particularly from cancer—may reflect the cumulative effects of structural barriers to preventive care. Evidence suggests that trans people AFAB have substantially lower participation in cervical (and other) cancer screening than cisgender women, and structural barriers related to how sex/gender is recorded in health systems (including reduced automatic screening invitations after gender marker changes) may exacerbate under-screening over time.^37^

Our study boasts several strengths and unique contributions. It is, to our knowledge, the largest population-based study on all-cause and cause-specific mortality risk among trans populations globally and the first to provide these national estimates for Australia. The use of whole-of-population administrative data, combined with high-quality linkage to mortality and cause-of-death registries, enabled precise identification of mortality outcomes and cause of death across the entire population, minimising loss to follow-up and measurement error. An additional strength is the selection of a comparator group drawn from individuals who had recently visited a GP. This reduces potential healthy-user bias by ensuring that both groups were engaged with primary care at baseline, thereby improving comparability in health-care contact and opportunities for diagnosis of health conditions. These features provide a robust foundation for generating reliable estimates of mortality risk and have important implications for social and health policy.

First, at the population level, our results suggest that efforts to reduce premature mortality among trans people should be tailored to address changing patterns of risk across the life course. This includes strengthening early detection and prevention efforts for cardiovascular disease and cancer in older populations, alongside sustained investment in mental health services. Further, suicide prevention strategies should reflect the elevated risks seen in both younger trans people and older trans people AFAB, who experience particularly elevated risks.

Unmet healthcare needs has been cited as a key issue among gender minorities internationally and in Australia.^38^ Evidence suggests that trans populations are more likely to experience fragmented care, are less likely to have continuous medication coverage, and have reduced uptake of cancer screening programs such as cervical, breast, and bowel cancer screening relative to the general population.^2^^,^^39^ Referral and monitoring of risk factors and chronic disease screening and management strategies should be prioritised in trans populations, commencing with a more thorough research investigation into barriers and facilitators influencing preventive health services uptake.

The elevated mortality risk for chronic conditions further suggests there may be a need for tailored clinical guidelines and targeted action to improve medical care in trans populations. Research suggests that trans people who visit providers with greater experience treating trans patients receive better continuity of care.^39^ Indeed, access to trans community health organisations alongside safe, accessible, and inclusive mainstream healthcare has been recognised as a key priority within Australia's recently announced 10-Year National Action Plan for the Health and Wellbeing of LGBTIQA + People (2025–2035; ‘the National Action Plan’).^40^ As a part of the National Action Plan, funding has been earmarked for LGBTIQA + Community Controlled Health Organisations and for training mainstream primary health providers. The extent to which these measures impact provider interactions with trans patients impact and continuity of care should be investigated in future work.

The excess mortality risk from external causes and suicide, suggests the need for urgent policy response to address causes of underlying mental health inequalities. Earlier research on drivers of mental health inequalities among trans populations highlights the critical importance of reducing discrimination and stigma, particularly at the institutional level.^41^^,^^42^ Australia has made sweeping legislative changes pertaining to the rights and protections for gender minorities in Australia over the past decade, including bolstering anti-discrimination protections and improved recognition of gender diversity in official identification documents.^43^ Yet progress remains uneven. For example, although a substantial body of research has shown that access to gender-affirming medical care is associated with better mental health outcomes, including psychological functioning, reduced symptoms of depression and suicidal ideation, and reduced mental health treatment among trans populations,^36^^,^^44^^,^^45^ there is inadequate and inequitable access in Australia. And this disproportionately impacts young people; for example, Australia's third most populous state recently restricted access to gender-affirming medical care for people under 18 years.^46^ Notwithstanding these legislative barriers to care, exposure to anti-trans rhetoric and discrimination has intensified in recent years^47^ and this is postulated to be a driver of widening mental health inequalities observed in trans Australians, particularly for young people.^3^^,^^48^ Alongside affirming youth-focused mental health support, policy responses should therefore acknowledge and address these structural factors.

Finally, our results reinforce the need to strengthen data infrastructure to better characterise the health needs of trans people, including those who are not accessing gender-affirming medical care. Embedding gender identity measures in population-level datasets, such as the Census,^49^ and continued investment in large-scale community-based surveys^50^ will be essential to monitor progress and guide interventions to reduce premature mortality among trans populations.

### Limitations

These results should be considered within the context of several limitations. In this paper, comparator populations are referred to as general population males and general population females, although we were unable to verify that these people were cisgender (i.e., not trans). While use of nationally linked administrative data provided unprecedented advantages in terms of sample size and statistical power, the trans populations identified herein were restricted to those who accessed subsidised gender-affirming hormone therapy. As such, our findings speak specifically to trans Australians who initiated gender-affirming hormone therapy, and caution is needed in generalising to all trans people. While there is no population level evidence, survey-based estimates suggest that 63% of non-binary Australians had or desired to use gender-affirming hormone therapy^51^ and this figure is higher (84%) for younger people (14–25 years).^52^

Our sample also excludes trans people who received other types of gender-affirming healthcare independent of hormone therapy, such as gender-affirming surgeries. However, available Australian evidence indicates that most people undergoing gender-affirming surgery are already on gender-affirming hormone therapy, or undertake hormones prior to surgery (>90%).^8^ Thus, while our sample does not capture all trans people receiving surgical care, it likely captures the large majority of those receiving gender-affirming medical interventions in Australia.

However, the extent to which this would bias our estimates is unclear. For instance, our sample likely excludes trans people who may not have access to subsidised care, such as recent migrants, or individuals experiencing other barriers to medical care, such as inadequate access or fears of discrimination. Groups with barriers to primary and preventive care may therefore be at higher risk of adverse health outcomes than trans people who were able to access hormone therapy. Supporting this interpretation, recent Australian work has shown that total healthcare costs decrease following initiation of gender-affirming hormone therapy.^53^ To the extent that healthcare utilisation and costs act as proxies for underlying health status, these findings reinforce the likelihood of potential health improvements associated with access to gender-affirming hormone therapy. This would suggest that our estimates may represent a conservative lower bound and further reinforces that the elevated mortality observed in this study should not be interpreted as being attributable to gender-affirming hormone therapy itself.

It is also important to note that there are multiple ways to affirm one's gender (e.g., socially), and gender-affirming medical care may not be a goal for many trans people. Mortality risks are therefore likely to differ across subgroups defined by social, medical, or no medical-related care. Future work understanding how mortality risks diverge within different subgroups of trans populations is warranted.

Further, despite fewer deaths among trans people AFAB, reflecting their younger age at treatment initiation, the accumulation of over 30,000 person-years of follow-up yielded a sufficient number of events relative to person-time to support asymptotic inference.^54^

Finally, while our theoretical framing draws on minority stress and weathering processes as key drivers of mortality inequalities, we acknowledge that broad ICD-10 groupings (e.g., cardiovascular disease) include heterogeneous causes, some of which are less directly linked to chronic stress pathways. These categories were retained to ensure adequate statistical power, but this may dilute associations for specific subcomponents.

### Conclusions

This nationwide cohort study provides the first population-based estimates of mortality risk for trans Australians relative to the general population. We find that trans people experience premature mortality relative to the general population, but underlying causes vary by sex at birth and across age groups. Deaths from external causes and suicide are most elevated among middle-aged and older trans people AFAB and among younger and middle-aged trans people AMAB. Cardiovascular and cancer account for much of the excess mortality observed in older trans people.

## Contributors

KS, BN, SB, and DP conceptualised the study and methodology. KS and BN acquired funding. KS conducted formal analysis and project administration. BN accessed and verified the data. KS wrote the first draft of the manuscript. Interpretations of the results were made by KS, SB, DP, and BN. All authors reviewed the analyses and drafts of this manuscript, approved its final version, and were responsible for the decision to submit the manuscript.

## Data sharing statement

Data is available upon request to the Australian Bureau of Statistics.

## Declaration of interests

KS is a cofounder, and serves on the committee, of LGBTQ+ Economists and Allies in the Asia–Pacific (LEAP).

## Footnotes

g The authors respectfully use the term ‘assigned at birth’ to refer to the common experiences of healthcare professionals observing the physical appearance and physiology of a newborn to make a value-laden judgement about what information to record on their birth certificate about their ‘sex’. In the literature, this phrase is also termed as ‘presumed at birth’ and ‘recorded at birth’. The authors acknowledge that sex is not relevant to gender. Sex-stratified mortality analyses are relevant to the present paper because gender-affirming hormone therapy treatment regimens differ by sex and the potential effects of these regimes on mortality may consequently differ.
