# Supplementary material for: All-cause and cause-specific mortality among transgender and gender diverse people: a nationwide cohort study in Australia
Source: Lancet Reg Health West Pac. 2026 Feb 9;67:101813. doi: 10.1016/j.lanwpc.2026.101813 (PMC12914191; doi:10.1016/j.lanwpc.2026.101813)
Supplement: Supplementary Material [file mmc1.docx]

# Supplementary Material

## ICD-10 codes

| **Cause of death** | | **ICD-10 codes** |
| --- | --- | --- |
| External | Suicide | X60–X84; Y87.0 |
|  | Alcohol-related causes | K70, K73–K74, F10, K85–K86, I42.6 |
|  | Drug and poisoning related deaths | X40–X49; Y10–Y15; Y45; Y47; Y49; I42.7 |
|  | Unintentional injuries | V01–V99; W00–W99; X00–X59; Y85–Y86 |
| Cancer | | C00–C97 |
| Cardiovascular | | ICD-10 Codes starting with ‘I’ |

## Unweighted descriptive characteristics

|  | **Trans people  AFAB (n=9,713)** | **General population females (n=10,282,651)** | **Trans people  AMAB (n=17,172)** | **General population  males (n=9,879,037)** |
| --- | --- | --- | --- | --- |
| Age entry, mean, years | 25.40 | 44.90 | 36.15 | 44.39 |
| Age entry, median, years | 22.00 | 42.75 | 27.92 | 42.67 |
| *Age group entry (years)* |  |  |  |  |
| 15-24, No. (%) | 6359 (65.47) | 1,682,439 (16.36) | 7,876 (40.71) | 1,586,639 (16.06) |
| 25-39, No. (%) | 2506 (25.8) | 2,945,396 (28.64) | 5,113 (26.43) | 2,858,575 (28.94) |
| 40-59, No. (%) | 637 (6.56) | 3,271,560 (31.82) | 3,406 (17.6) | 3,278,683 (33.19) |
| 60+, No. (%) | 211 (2.17) | 2,383,256 (23.18) | 2,952 (15.26) | 2,155,140 (21.82) |
| Follow-up, mean, years | 3.33 | 10.29 | 4.53 | 9.96 |
| Follow-up, median, years | 2.83 | 11.42 | 3.67 | 11.25 |
| Person-years, total | 32,342 | 105,794,750 | 88,072 | 98,346,555 |
| Death – any cause, No. (%) | 59 (0.61) | 840,418 (8.17) | 1,066 (5.51) | 908,100 (9.19) |
| Death – external, No. (%) | 22 (0.23) | 46,623 (0.45) | 118 (0.61) | 83,135 (0.84) |
| Death – suicide, No. (%) | 18 (0.19) | 7693 (0.07) | 64 (0.33) | 23,215 (0.23) |
| Death – cardiovascular disease, No. (%) | 10 (0.1) | 229,154 (2.23) | 159 (0.82) | 230,003 (2.33) |
| Death – cancer, No. (%) | 16 (0.16) | 226,069 (2.2) | 487 (2.52) | 291,483 (2.95) |

Notes: Trans=Trans and gender diverse; AMAB=Assigned male at birth; AFAB=Assigned female at birth. Unweighted sample.

## Results when weighting general population to be similar to trans population based on age, month, and electorate at treatment initiation

|  | **Trans people AFAB  compared to general population females** | | | | | **Trans people AMAB  compared to general population males** | | | | |
| --- | --- | --- | --- | --- | --- | --- | --- | --- | --- | --- |
|  | **Full sample** | **15-24 years** | **25-39 years** | **40-59 years** | **60+ years** | **Full sample** | **15-24 years** | **25-39 years** | **40-59 years** | **60+ years** |
| All-cause | 9.57*** [7.28;12.58] | 4.62*** [2.33;9.14] | 7.30*** [3.84;13.89] | 3.77*** [2.03;6.98] | 2.41*** [1.58;3.66] | 4.72*** [4.44;5.03] | 2.84*** [1.99;4.05] | 3.40*** [2.46;4.71] | 2.19*** [1.83;2.62] | 2.51*** [2.34;2.70] |
| Cardiovascular disease | 15.24*** [7.85;29.57] | N/A | 18.84*** [5.55;64.01] | N/A | 3.31** [1.52;7.21] | 3.24*** [2.76;3.80] | 2.15 [0.37;12.56] | 2.12 [0.68;6.58] | 1.81** [1.20;2.74] | 1.53*** [1.27;1.83] |
| Cancer | 7.71*** [4.66;12.75] | N/A | N/A | 3.34** [1.45;7.72] | 2.04* [1.06;3.92] | 6.12*** [5.58;6.72] | 1.33 [0.21;8.25] | 1.10 [0.27;4.48] | 2.29*** [1.73;3.02] | 3.43*** [3.10;3.80] |
| External | 9.83*** [6.16;15.69] | 8.02*** [3.89;16.56] | 8.50*** [3.38;21.35] | 10.00*** [3.12;32.03] | N/A | 3.12*** [2.59;3.76] | 2.89*** [1.95;4.28] | 4.67*** [3.20;6.81] | 2.41*** [1.63;3.58] | 1.45 [0.97;2.17] |
| Suicide | 12.75*** [7.38;22.02] | 11.35*** [4.75;27.10] | 11.44*** [4.42;29.62] | 19.13*** [4.47;81.86] | N/A | 4.27*** [3.31;5.51] | 4.25*** [2.72;6.64] | 6.50*** [4.11;10.29] | 2.84*** [1.55;5.19] | 1.95 [0.82;4.67] |

Notes: Trans=Trans and gender diverse; AMAB=Assigned male at birth; AFAB=Assigned female at birth. General population are those who first visited a GP at the same time and are weighted using inverse probability weights so that distribution of observed covariates pertaining to ‘age and month-year of start’ and ‘electorate at treatment start’ is balanced between groups. N/A=not applicable (few deaths in the trans population). *** p<0.001 ** p<0.05 * p<0.1

## Results when comparing trans population to opposite sex at birth

|  | **Trans people AFAB  compared to general population males** | | | | | **Trans people AMAB  compared to general population females** | | | | |
| --- | --- | --- | --- | --- | --- | --- | --- | --- | --- | --- |
|  | **Full sample** | **15-24 years** | **25-39 years** | **40-59 years** | **60+ years** | **Full sample** | **15-24 years** | **25-39 years** | **40-59 years** | **60+ years** |
| All-cause | 3.53*** [2.71;4.59] | 1.25 [0.66;2.39] | 2.72** [1.46;5.06] | 1.91* [1.04;3.51] | 1.40 [0.92;2.15] | 4.22*** [3.97;4.49] | 7.33*** [5.08;10.60] | 7.02*** [5.07;9.71] | 3.14*** [2.63;3.76] | 2.07*** [1.93;2.22] |
| Cardiovascular disease | 6.62*** [3.53;12.43] | N/A | 7.30*** [2.30;23.17] | N/A | 1.96 [0.89;4.32] | 2.54*** [2.17;2.99] | 3.38 [0.54;21.19] | 4.59** [1.46;14.38] | 4.49*** [2.97;6.79] | 1.10 [0.92;1.32] |
| Cancer | 5.73*** [3.45;9.51] | N/A | N/A | 2.57* [1.11;5.91] | 1.18 [0.61;2.29] | 5.89*** [5.36;6.46] | 1.57 [0.25;9.90] | 0.96 [0.24;3.92] | 2.35*** [1.79;3.09] | 3.82*** [3.45;4.23] |
| External | 2.37*** [1.55;3.63] | 1.90 [0.99;3.65] | 2.31 [0.97;5.52] | 2.48 [0.79;7.85] | N/A | 6.75*** [5.59;8.15] | 9.82*** [6.48;14.88] | 16.58*** [11.27;24.38] | 6.65*** [4.49;9.86] | 1.49 [0.99;2.22] |
| Suicide | 3.90*** [2.42;6.28] | 3.12** [1.48;6.56] | 4.52*** [1.87;10.91] | 4.91* [1.20;20.09] | N/A | 14.01*** [10.74;18.28] | 13.35*** [8.27;21.54] | 20.63*** [12.78;33.32] | 9.90*** [5.40;18.18] | 5.41*** [2.12;13.80] |

Notes: Trans=Trans and gender diverse; AMAB=Assigned male at birth; AFAB=Assigned female at birth. General population are those who first visited a GP at the same time and are weighted using inverse probability weights so that distribution of observed covariates pertaining to ‘age and month-year of start’ and ‘electorate at treatment start’ is balanced between groups. N/A=not applicable (few deaths in the trans population). *** p<0.001 ** p<0.05 * p<0.1
